# Phrases in noise test (PINT) Brazil: effectiveness of the test in children with hearing loss

**DOI:** 10.1016/j.bjorl.2019.07.010

**Published:** 2019-09-18

**Authors:** Regina Tangerino de Souza Jacob, Camila Oliveira e Souza, Bruna Camilo Rosa, Larissa Germiniani dos Santos, Elaine Cristina Moreto Paccola, Bianca Gonçalves Alvarenga, José Roberto Pereira Lauris

**Affiliations:** a Universidade de São Paulo (USP), Faculdade de Odontologia de Bauru (FOB), Departamento de Fonoaudiologia, Bauru, SP, Brazil; b Universidade de São Paulo (USP), Hospital de Reabilitação de Anomalias Craniofaciais (HRAC), Divisão de Saúde Auditiva, Bauru, SP, Brazil; c Universidade de São Paulo (USP), Faculdade de Odontologia de Bauru (FOB), Departamento de Odontopediatria, Ortodontia e Saúde Coletiva, Bauru, SP, Brazil

**Keywords:** Speech perception, Noise, Signal to noise ratio, Hearing tests, Child

## Abstract

**Introduction:**

One of the main implications of hearing impairment is the difficulty in perceiving speech sounds, especially in noisy environments. Thus, the frequency-modulated system is considered an important educational tool for children with hearing impairment because it improves speech perception in acoustically-unfavorable environments, such as in the classroom. The assessment of speech perception in noise is included in the verification protocol of this device.

**Objectives:**

To verify the effectiveness of the phrases in noise test Brazil in children with hearing impairment using an frequency-modulated system.

**Methods:**

This was a cross-sectional cohort study. The sample included 40 children, aged 4 years to 11 years and 11 months old, divided into 4 groups: (1) 10 normal hearing children; (2) 13 children with hearing aids and frequency-modulated system; (3) 12 children using cochlear implant and fitted with the frequency-modulated system; and (4) 5 children diagnosed with auditory neuropathy spectrum disorder, fitted with hearing aids and/or cochlear implant and with the frequency-modulated system. The phrases in noise test Brazil was used to evaluate speech perception in noise under the conditions with and without the frequency-modulated system. For the statistical analysis of the data, a significance level of 5% (*p* < 0.05) was adopted.

**Results:**

There was a significant difference between the groups when they were evaluated with the frequency-modulated system. The test was also validated through concurrent and convergent validation measures. Phrases in noise test Brazil is a viable option for monitoring auditory performance in noise in different groups of children with hearing impairmen.

**Conclusion:**

Phrases in noise test Brazil was effective in assessing speech perception in noise and may contribute to the improvement of the indication, fitting and follow-up protocols for the frequency-modulated system use.

## Introduction

One of the main implications of hearing impairment (HI) is the difficulty in perceiving speech sounds.^1^ In cases of congenital hearing loss, HI can result in delayed or even impaired oral language acquisition.

Currently, the technology embedded in Hearing Aids (HAs) and cochlear implant (CI) speech processors and bone conduction hearing aids allows access to speech perception, modifying and enhancing oral language acquisition with the help of speech therapy. In Brazil, these devices can be acquired in accredited Hearing Health Services, following criteria published by the Brazilian Unified Health System (SUS, *Sistema Único de Saúde*).^2^, ^3^

In the Hearing Health area, the most recent addition to the list of orthoses and prostheses provided by SUS is the Frequency-Modulated System (FM System).^4^ The FM System is a remote microphone consisting of a transmitter and a receiver, which aims to capture the speech signal and transmit it directly to the listening device without the need for connecting wires.^5^

Thus, the FM system is considered an important educational tool for children with HI,^6^ because it facilitates improvement in speech perception in acoustically unfavorable environments, such as the classroom.^7^

The American Academy of Audiology has developed a best practice guide^8^ for the evaluation of remote microphones. The assessment of speech perception in noise is included in the test battery evaluating this device. The Brazilian Hearing in Noise Test (HINT) is currently used in Brazil,^9^ as well as the List of Sentences in Portuguese (LSP) test,^10^ structured for the adult population.^11^

Due to the scarcity of speech perception in noise tests for young children, the Phrases in Noise Test (PINT) was developed for children with CI,^12^ the objective of which is to obtain the child's speech recognition threshold in noise without the influence of variables related to the receptive vocabulary level or the speaker’s intelligibility of speech production. In 2015,^13^ it was culturally adapted to Brazilian Portuguese and its applicability was added to the evaluation of FM systems in children aged four years and older.^14^

Therefore, this study addresses the validation process of the PINT Brazil^13^, ^14^ in different populations of children with HI. Four groups were selected: (1) Control Group of 10 normal hearing children (normal hearing); (2) 13 children fitted with HA and FM system; (3) 12 children using CI and fitted with FM system; and (4) 5 children diagnosed with Auditory Neuropathy Spectrum Disorder (ANSD), fitted with HA and/or CI and with the FM system. Thus, the present study aimed to verify the effectiveness of the speech perception in noise PINT Brazil^13^, ^14^ in children with HI using the FM system.

## Methods

This is a cross-sectional study carried out at the Speech-Language Pathology Clinic of Faculdade de Odontologia de Bauru, Universidade de São Paulo (FOB/USP) and the Hearing Health Division of the Craniofacial Anomalies Rehabilitation Hospital, Universidade de São Paulo (HRAC/USP), after approval by the Research Ethics Committee (Certificate of Presentation for Ethical Appreciation (CAAE) n. 17699713.9.0000.5417, CAAE n. 56422116.8.3001.5441 and CAAE n. 62481816.2.0000.541), from May 2014 to August 2016. All participants were instructed on the research objectives and invited to sign the Informed Consent Form with their guardians. All participants were instructed regarding the study objectives and invited to sign the Free and Informed Consent form with their parents/guardians.

The following inclusion criteria were established: (a) Having a diagnosis: (1) normal hearing children for the control group, (2) children with moderate to severe sensorineural HI, fitted with HA, (3) children with severe to profound sensorineural HI, users of CI and (4) children diagnosed with ANSD, fitted with HA and/or CI; (b) users of the FM system; c) Aged 4 years up to 11 years and 11 months; d) being enrolled in elementary school.^4^

Thus, the convenience sample consisted of 40 children, aged 4 years up to 11 years and 11 months of age, regularly enrolled in the FOB/USP and HRAC/USP hearing health services. The sample was divided into four groups, as follows: (1) 10 normal hearing children; (2) 13 children fitted with HAs and FM system; (3) 12 children using CI and users of the FM system; and (4) 5 children with ANSD, fitted with HAs and/or CI and users of the FM system.

## Tools and procedures

### Evaluation of speech perception in noise

The speech perception in noise test was performed in an acoustically treated booth. A two-channel audiometer, model Astera AS by Madsen, and a two-speaker amplification system were used to present speech and noise stimuli in a free field, with an incidence angle at 0° (zero degree) azimuth and 180° (one hundred and eighty degrees) azimuth respectively, aiming to simulate the classroom environment.^8^

**Figure 1:**
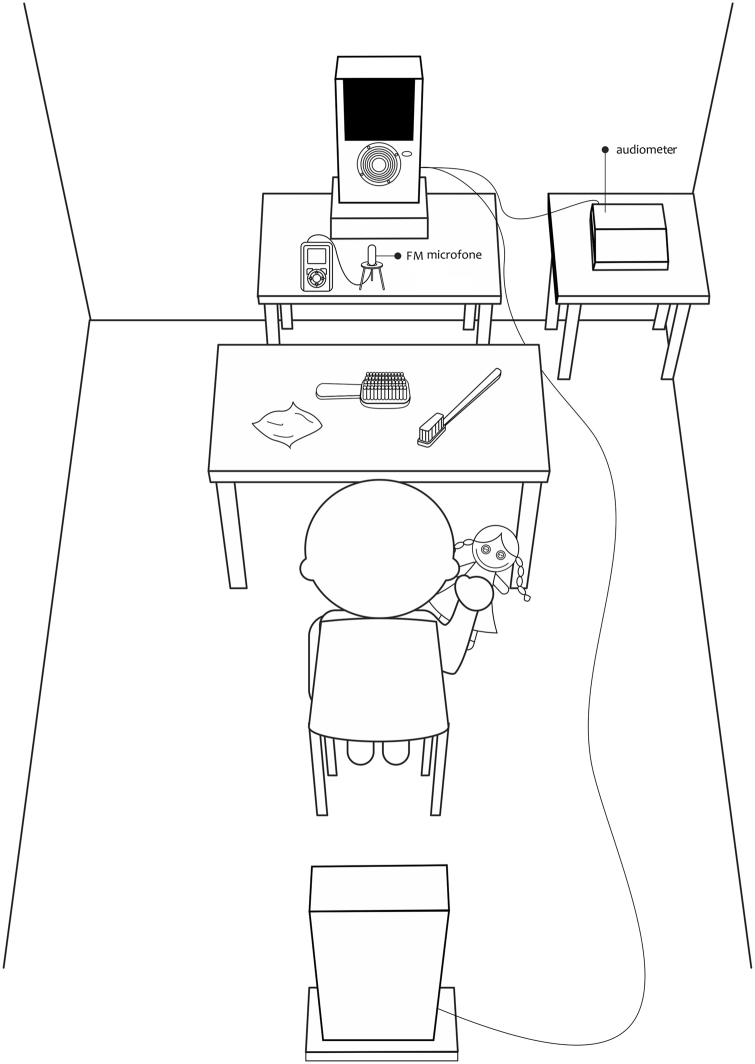
Application scenario of the PINT Brazil test.

The participants were seated in a chair in the middle of the booth, one meter away from each speaker. The FM system was placed at a distance of approximately 22 cm below the speaker that presented the speech stimulus. The test environment is described in Fig. 1.

The Phrases in Noise Test (PINT) Brazil^13^, ^14^ was used. The PINT^12^, ^13^, ^14^ uses the concept of threshold that estimates 50% of the correct sentence thresholds in the presence of adaptive noise levels, that is, varying between rising and falling intensities.

The test consists of 10 simple-order sentences referring to body parts (closed-set auditory recognition). Six test lists were created in the Brazilian Portuguese version, and each sentence is repeated twice per list in a pseudorandomized way. The test begins with a Signal/Noise ratio (SNR) of +15 dB SNR and the SNR decreases to −12 dB SNR; the test continues with a −12 dB SNR that progressively increases to +15 dB SNR. The speech signal is presented at a fixed intensity of 60 dB and the noise intensity varyies in an adaptive manner.

The noise in the PINT Brazil^13^, ^14^ was created by recording environmental noises in four elementary school classrooms during the class period. Samples of conversation between children and the movement of chairs and papers being leafed through were edited through specific audio editing programs, in order to reduce the amplitude of modulation between the recordings but maintain the spectral characteristics of classroom noise. The four samples were combined into a single four-minute wave, and the final sample is three minutes and two seconds long due to the exclusion of other noises, such as falling chairs and closing doors. The final noise sample was matched to the long term by the root-mean-square of the signal or Room Mean Squared (RMS) of the sentences, making it effective for masking the speech signal.

Before the PINT Brazil was applied^13^, ^14^ training was performed, in which 10 sentences were presented in the absence of noise, and 10 sentences at +15 dB SNR (training list). After each sentence was presented, the child was asked to listen to the sentence, understand it and perform the action requested on a doll. The test was started with adaptive noise only when the child was able to recognize 100% of sentences without noise and at +15 dB SNR. A table was placed in front of the child with the doll and the support objects (toothbrush, comb and towel).

The list presentation was performed in the following situations: (a) Only with the hearing device (HA or CI) and then; (b) With the hearing device paired with the FM System, in random order of presentation using the Latin square design.

**Figure 2:**
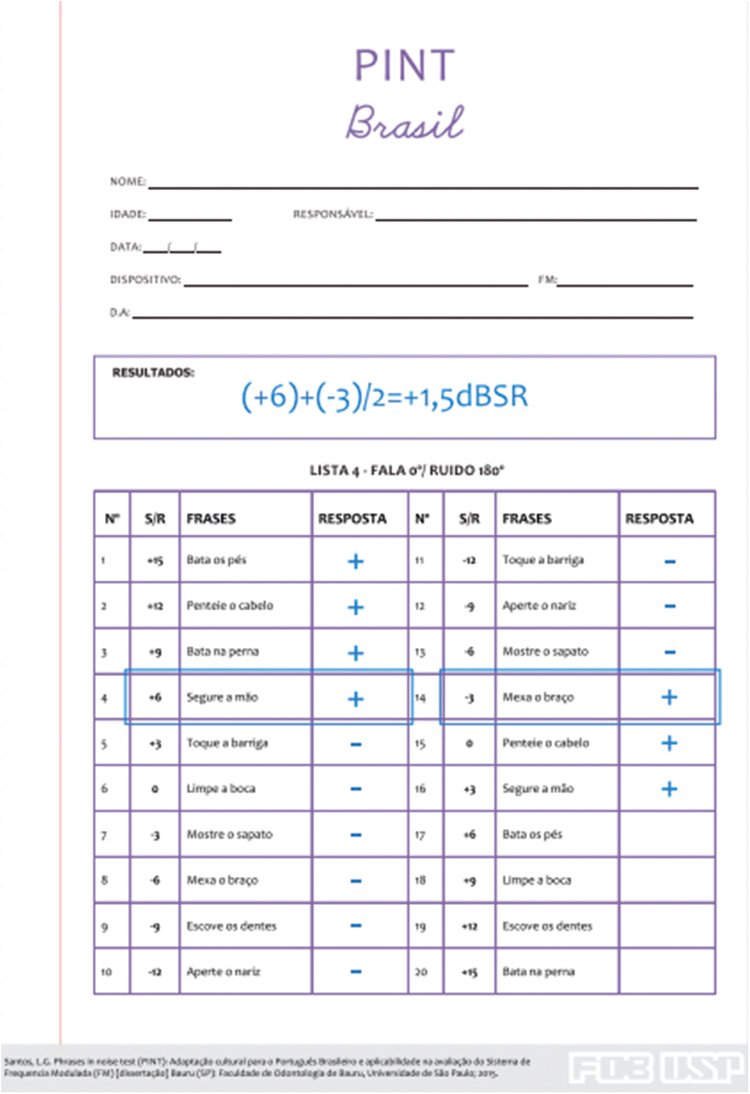
Answer sheet and score example for PINT Brazil. Source: Santos LG, Schafer EC, Thibodeau LM, Jacob RTS. The Brazilian Phrases in Noise Test (PINT Brazil). Journal of Educational, Pediatric and (Re)Habilitative Audiology (JEPRA). 2017;23:1-8. Reproduced with permission of the authors.

The answers were noted on the response form (Fig. 2) and the obtained results were determined by the speech recognition in noise threshold (dB SNR) according to the test score rules.^12^, ^13^, ^14^ The dB SNR threshold is determined by the mean of the scores: (1) at the decreasing intensity, the last correct answer is considered followed by two incorrect answers, and at the rising intensity, the first correct answer is considered followed by two more consecutive correct answers. If the child could not present three consecutive correct answers at the rising intensity, the +15 dB SNR value was considered. In case of 100% correct answers for all teste sentences, or just one incorrect sentence throughout the test, the threshold of -12dBSNR was adopted.

The test is interrupted when the child obtains three consecutive correct answers at the rising intensity of the answer form.

### Statistical analysis

The data were submitted to the Kolmogorov-Smirnov normality criterion. Intragroup comparisons were performed by paired *t*-test and intergroup comparisons by *t*-test for independent groups. A significance level of 5% (*p* < 0.05) was used for all tests.

The ANOVA hypothesis test was performed to calculate the sample size. The parameters considered for the test were the difference (Δ) between the situations WITH and WITHOUT the FM system after grouping of the four levels, which resulted in a mean difference of 2.65 and a standard deviation of 2.84. Considering a test power (K) of 80%, a chance of Type II error (β) of 20% and a significance level (α) of 5%, the estimated sample size comprised 27 participants.

## Results

### SNR (signal-to-noise ratio)

**Figure 3:**
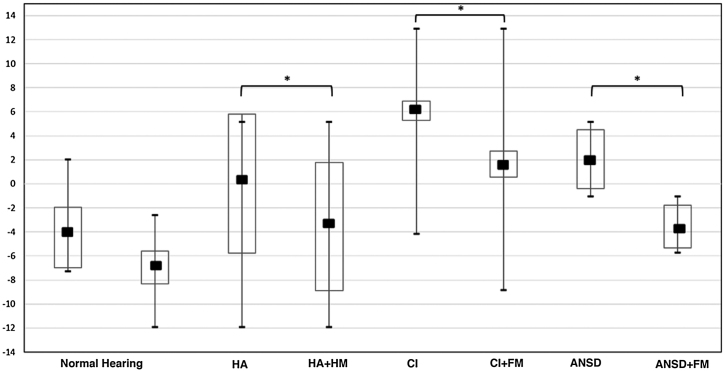
Box-plot chart showing the comparison of the mean (dB SNR) between the control group and the situations WITHOUT and WITH the FM System of the group of children with HA, CI and ANSD. HA: Hearing Aid; FM: FM System; CI: Cochlear Implant; ANSD: Auditory Neuropathy Spectrum Disorder. * p<0.05 is statistically significant.

The box-plot graph (Fig. 3) shows the comparative analysis of the control group (1st and 2nd situations) and of the situations WITHOUT and WITH the FM system in the group of children using HA, CI and those diagnosed with ANSD.

Table 1 shows the descriptive values of the subjects’ performance (dB SNR) for the PINT Brazil^13^, ^14^ and the comparison between all groups evaluated in the situations WITHOUT and WITH the FM System.

**Table 1:** Comparison of the mean signal-to-noise ratio (dB SNR) between all groups evaluated under the conditions WITH and WITHOUT FM System.

Group 1 × Group 2	n	Mean (dB SNR)	SD (dB SNR)	*p*
Normal hearing × HA	10/13	−6.0/−0.3	1.3/5.4	0.00^a^
Normal hearing × HA FM	10/13	−6.0/−3.9	1.3/5.1	0.23
HA × CI	13/12	−0.3/4.4	5.4/4.2	0.03^a^
HA FM × CI	13/12	−3.9/4.4	5.1/4.2	0.00^a^
HA × CI FM	13/12	−0.3/1.0	5.4/5.2	0.56
HA FM × IC FM	13/12	−3.9/1.0	5.1/5.2	0.03^a^
HA × ANSD	13/05	−0.3/1.5	5.4/2.4	0.50
HA FM × ANSD FM	13/05	−3.9/−3.9	5.1/1.7	0.99
ANSD × Normal hearing	05/10	1.5/−6.0	2.4/1.3	0.00^a^
ANSD FM × Normal hearing	05/10	−3.9/−6.0	1.7/1.3	0.02^a^
ANSD × CI	05/12	1.5/4.4	2.4/4.2	0.18
ANSD FM × CI	05/12	−3.9/4.4	1.7/4.2	0.00^a^
ANSD × CI FM	05/12	1.5/1.0	2.4/5.2	0.84
ANSD FM × CI FM	05/12	−3.9/1.0	1.7/5.2	0.06

n, Number of children per group; HA, Hearing Aid; FM, Frequency-Modulated System; CI, Cochlear Implant; ANSD, Auditory Neuropathy Spectrum Disorder.

a *p* < 0.05 is statistically significant.

Table 2 shows the series of cases and the SNR values found in the group of children using CIs.

**Table 2:** Description of the series and the value of SNR ratios (dB SNR) found in the group of children using CI.

n	Gender	Age	1CI (dB SNR)	1CI FM (dB SNR)	2CI (dB SNR)	2CI FM in 1CI (dB SNR)
1	M	9	3	−9	6	−7.5
2	F	9	7.5	0	6	4.5
3	M	8	3	0	3	1.5
4	M	4	9	6	4.5	−4.5
5	F	4	9	−1.5	9	1.5
6	F	8	6	1.5	6	4.5
7	M	10	9	−1.5	4.5	4.5
8	M	9	4.5	4.5	−4.5	−3
9	F	8	12	−1.5	0	−1.5
10	F	8	7.5	3	4.5	3
11	F	7	6	10.5	12	12
12	M	8	0	1.5	1.5	−3
Mean	‒	7.6	6.4	1.1	4.4	1.0

n, Number of children; 1CI, Monaural Cochlear Implant; 1CIFM, Monaural Cochlear Implant, paired to the FM System; 2CI, bilateral cochlear implant; 2CI FM, Bilateral Cochlear Implant, with the first CI paired to the FM System.

Table 3 shows the comparison between the PINT Brazil and HINT Brazil tests, with the aim of attaining convergent validation for the PINT Brazil.

**Table 3:** Comparative data of the values of the SNR ratios of the PINT Brazil × HINT Brazil tests for convergent validation.

	n	Mean	SD	*p*
PINT Brazil	10	−6.0	1.3	0.80
HINT Brazil	21	−6.2	2.3	

n, Number of children; SD, standard deviation; *p* < 0.05 is statistically significant.

## Discussion

According to the literature, individuals with hearing loss of cochlear origin show greater difficulty in speech perception in noisy environments and need +15 dB to +20 dB for the SNR to be favorable for speech perception.^15^, ^16^, ^17^ This fact is shown in Fig. 3, which shows a satisfactory performance of speech perception in noise of normal-hearing children in comparison to the other assessed groups.

Statistically significant differences (*p* < 0.05) can be observed when comparing the situations with and without the FM System in the group of children using PSAD, CI and with ANSD, consistent with the consensus in the literature that the FM System promotes the improvement of speech perception in noise.^18^, ^19^, ^20^

Individuals with moderate to severe HI, under the condition of HA WITH the FM system, showed similar performance to normal-hearing children and there was no statistically significant difference between the two groups (Table 1). However, the group did not perform as well when evaluated WITHOUT the FM system, which demonstrates the importance of using the remote microphone in the classroom.

A statistically significant difference can be observed in the group of children using CI in the comparative analysis of their variables (Fig. 3), under the conditions WITH and WITHOUT the FM system. The use of the binaural resource in children with HI, either through the use of bimodal input technology (HA + CI) or bilateral input (2CI), helps in speech recognition performance in the presence of noise when compared to the use of monoaural resource (1CI),^12^, ^21^, ^22^, ^23^, ^24^ as observed in Table 2. In the assessed group, there was a 2 dB improvement for bilateral input.

In Table 2, the age at the first CI ranged from 2.5 to 8.2 years and the second CI from 9 months to 3.3 years; with the mean difference in auditory age from the first CI to the second CI being 4.7 years, which may explain the obtained result. It is worth mentioning that children make effective use of the FM system only with the first CI.

As for the children diagnosed with ANSD, there was no statistically significant difference when comparing the results of children using CI in the situation WITH the FM system (Table 1), corroborating the studies^25^, ^26^, ^27^ demonstrating that after the CI surgery, individuals with ANSD improved their auditory skills and performed similarly to children with sensorineural hearing loss with a CI, with significant improvement in speech perception skills. According to Walker (2016),^28^ the fitting of the HA and/or CI and the FM system are the recommended alternatives as part of the intervention process in children diagnosed with ANSD.

The limitation found in this study was the small number of participants with ANSD. However, the occurrence of this pathology varies from 0.3% to 1.3% in the population using audiological clinical services, and 12–14% have already been diagnosed with severe to profound hearing loss of cochlear origin.^29^ Other studies indicate that among children with congenital HI, 2–15% are diagnosed with ANSD, with variable clinical presentations.^30^, ^31^, ^32^

In this group, different results were obtained among the participants, which can be explained by the factors that influence the performance of hearing and language skills of children with HI, such as: duration of sensory deprivation, type of device and time of use, family permeability in the therapeutic process and the speech sound coding strategy.

Concurrent and convergent validation measures were performed to validate the PINT Brazil. Concurrent validity, which expects the test to detect specific differences predicted in distinct groups, ^33^ was confirmed in the present study using two different methods: (a) Comparing normal-hearing children with children with HI and (b) comparing children with HI under the conditions WITH and WITHOUT the FM system. The group of normal-hearing children showed better results at the PINT Brazil than children using CI, WITHOUT or WITH the FM system. The SNR always has been shown to be more negative in the list of normal-hearing children than for children with HI under any assessed condition.

For convergent validation, where two measures need to be similar to each other,^33^ the results of the PINT Brazil were compared with the SNR values obtained at the HINT Brazil, with the noise being presented at 180° azimuth (RT position) in the study by Jacob et al.^11^ According to Table 3, data from the 10 normal-hearing children in the present study supported the convergent validity,^22^ as there was no significant difference in the results obtained between two different tests through an adaptive procedure, which evaluated 50% of the speech perception threshold in noise in normal-hearing children, that is, between the HINT Brazil^11^ and the PINT Brazil.^13^, ^14^ It is also possible to observe that there is no statistically significant difference (*p* < 0.05) between the two tests.

Therefore, the PINT Brazil^13^, ^14^ is a viable option for monitoring auditory performance in noise in different groups of children with HI.

## Conclusion

The PINT Brazil^13^, ^14^ was effective in assessing speech perception in noise and may contribute to the improvement of the FM system indication, fitting and follow-up protocols.

## Funding

This study received support from 10.13039/501100002322Coordenação de Aperfeiçoamento de Pessoal de Nível Superior (CAPES) - Finance Code 001, Conselho Nacional de Desenvolvimento Científico e Tecnológico (10.13039/501100003593CNPq, Process n. 484154/2013-3), Fundação de Amparo à Pesquisa do Estado de São Paulo (10.13039/501100001807FAPESP, Process n. 13/10283-0) and the Unified Scholarship Program of Universidade de São Paulo.

## Conflicts of interest

The authors declare no conflicts of interest.
